# Influence of Different Filler Systems on the Thermal Conductivity and Mechanical Properties of Thermosets

**DOI:** 10.3390/polym16202917

**Published:** 2024-10-17

**Authors:** Uta Rösel, Dietmar Drummer

**Affiliations:** Institute of Polymer Technology, Friedrich-Alexander-University Erlangen-Nuremberg, 91058 Erlangen, Germany; dietmar.drummer@fau.de

**Keywords:** thermal conductivity, thermosets, isolation of electronic components

## Abstract

The changing demands in terms of the compactness and performance of electronic devices increase the role of thermal management within this application field. Because polymers and especially thermosets have a low thermal conductivity, filler systems have to be used to improve their performance and make thermosets suitable for applications. So far, several factors influencing thermal conductivity have been defined; however, the combined investigation of mechanical properties as another important value in terms of applications has not been realized. Therefore, this paper analyzes thermal conductivity based on the orientation of fillers and a contact path as well as mechanical properties in different material systems. In addition to the matrix material, the filler type and grade and the geometry and size were varied. It was shown that boron nitride provides the best values in terms of thermal conductivity after orientation along the flow path is realized. An aluminum silicate was defined as the best filler system in terms of mechanical properties. However, the boron nitride was found to reveal the greatest potential in terms of applications, since the mechanical properties are likely to be increased by the usage of finishing additives such as silane.

## 1. Introduction

### 1.1. Application Fields and Possible Extensions

With the increasing compactness and performance of modern electronic devices, such as power electronics and electric motors, thermal management plays an increasing role in terms of the construction of samples within these application fields. In addition, there is more heat, which is generated by higher-power devices and samples, which allow greater operating temperatures in applications such as silicon carbide chips. Within silicon carbide semiconductors, the possible temperature in an application has increased from 90 °C [1] to 250 °C within the last 20 years [2]. The required heat resistance in applications, such as the encapsulation of electronics or electric motors, has led to the usage of thermosets, mainly epoxy resin [3]. The requirements for these materials are not only high thermal conductivity, but further, high electric isolation. Since polymers mostly have low electric and thermal conductivity, fillers are needed to satiate both demands. Thermosets reveal the possibility of increasing the filler content relative to thermoplastics since the viscosity behavior is different and reaches a smaller value. 

### 1.2. Thermal Conductivity of Thermoset-Based Compounds

Polymers mainly transport heat by the propagation of elastic waves through covalent bonds along one macromolecule. Further, secondary valence bonds like van der Waals forces allow the transfer of heat along molecules [4]. Transportation within a molecule is more effective than the method in between molecules. Thermal conductivity also increases with molecular mass, as the number of chain ends decreases, and thus does the number of van der Waals bonds. According to [5], thermal conductivity is limited relative to the increasing molecular mass, which depends on the chemical structure of a polymer [5].

**Figure 1:**
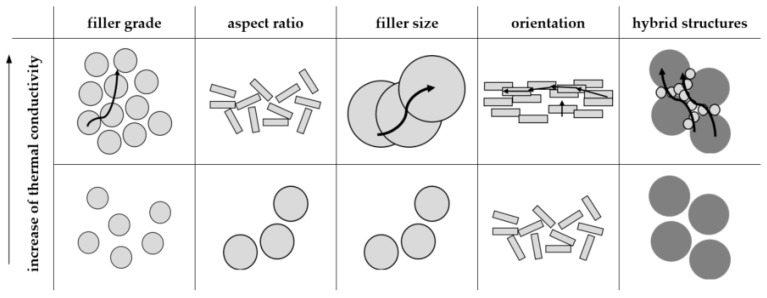
Different methods of improving thermal conductivity in terms of a compound system, according to [4,5,8,9].

To increase the possible thermal conductivity within polymers, filler systems must be used. To maintain an electric isolating behavior in thermal conductivity, mineral or ceramic filler types are likely to be used [6]. Most commonly, the thermal conductivity within polymers is increased by filler systems based on boron nitride (BN), zinc oxide (ZnO), or graphite. The initial thermal conductivity of BN is ten times larger than that of ZnO [3]. In addition to the initial properties of the filler, several factors have been defined, which reveal an impact on the thermal conductivity of the compound. According to [7], the matrix material, the filler size, the size distribution and the geometry are factors influencing thermal conductivity. Since the number of contact points is an essential determiner of thermal conductivity, the filler content plays an important role too. In [8], it was proven that an increase in filler grade together with a higher aspect ratio results in a greater improvement in thermal conductivity than with lower aspect ratios [8]. In general, platelet- or rod-shaped fillers lead to a higher thermal conductivity than spherical ones [4]. However, these geometries mostly lead to anisotropic properties throughout the sample, since thermal conductivity is mostly only high in one direction, e.g., along the fiber axis. In terms of the probability of contact points and large contact areas, larger particles are preferred, especially if they have an orientation with a conductive path [5]. It has to be taken into account that size is only one factor affecting improvement in thermal conductivity. Although larger fillers increase the number of contact points in case of a direct tangency, the average distance between larger fillers is higher than that between smaller ones with the same filler grade. This considered, the usage of large fillers may not improve thermal conductivity, since the other aspects have to be taken into account as well. In addition, hybrid systems, where small and large particle sizes are combined, reduce the space between fillers and, with that, improve the thermal conductivity [9]. Figure 1 summarizes the possibilities of increasing the contact areas and, with that, the thermal conductivity; the direct path along the fillers is highlighted with a line. 

In terms of epoxy resin, the highest thermal conductivity was reached in the range between 10 and 20 W∙m^−1^∙K^−1^. In [10], a thermal conductivity of 10.98 W∙m^−1^∙K^−1^ was proven based on the filler system aluminum nitride with a filler grade of 60 vol.-% and the surface modification of silane in an epoxy resin. By the combination of different anisotropic boron nitride particles, ref. [11] achieved a thermal conductivity of 19.0 W∙m^−1^∙K^−1^ again based on epoxy. 

To understand the influence of the fillers within the matrix materials, first attempts were made in terms of simulating the interaction between one filler and the surrounding as shown in [12]. 

### 1.3. Aim of This Paper

In terms of the changing demands within electronic devices, the thermal conductivity of polymer-based or, more precisely, thermoset-based compounds plays an important role in maintaining and expanding the application possibilities. So far, different influencing factors regarding the thermal conductivity in polymers have been investigated. In terms of applications in the field of electronic devices, besides the thermal conductivity, the electric isolation and the mechanical properties play an important role too. Further, the orientation of the fillers and, with that, the possibility of realizing a contact path and a high thermal conductivity are not fully understood within thermosets. Therefore, this paper investigates both aspects of high thermal conductivity along the orientation of fillers and sufficient mechanical properties. Taking electronic devices into account, typical materials for the encapsulation of electrics are epoxy resins. According to typical material systems, such as EP 3162 E (Raschig GmbH, Ludwigshafen, Germany) and their manufacturer’s specification, the following values in terms of the mechanical properties were defined as industrial standards within this paper: an E-Modulus E_t_ of at least 17.4 GPa, a tensile strength σ_m_ of 76.7 MPa and an elongation at break ε_m_ of 0.45%. 

The paper investigates four different filler systems without hybrid structures and two matrix materials as well as filler grades and their impact on both the thermal conductivity and mechanical properties. Mainly, a material system shall be defined where both aspects reach the industrial standard at least. 

## 2. Materials and Methods

### 2.1. Materials

The matrix materials in these experiments were two thermoset types: an epoxy resin (EP) of the type Epoxidur EP 368/1 (Raschig GmbH, Ludwigshafen, Germany) and a phenolic resin (PF) of the type EPF 87120 (Raschig GmbH, Ludwigshafen, Germany). Both thermosets are a pre-mixture of resin, hardener, catalyst and, in terms of EP, carbon black pigments with the exact formulation being confidential. Table 1 shows the specification of the matrix material in terms of the density δ, heat capacity c (based on own measurements) and thermal conductivity λ as well as molding shrinkage (longitudinal or longwise) (based on manufacturer specification). 

**Figure 2:**
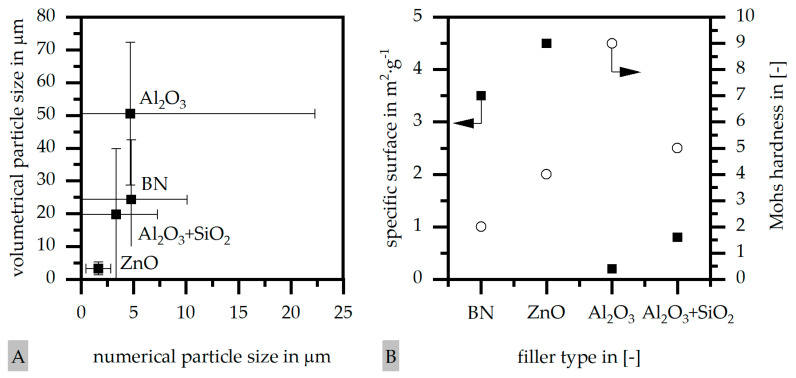
Specification of the filler systems including the mean particle size (numerical, volumetric) (**A**), the specific surface area (**B**) (own measurements) and the Mohs hardness (**B**) (manufacturer specification).

In the experiments, four different filler systems were evaluated. Besides the difference in thermal conductivity and density, the fillers vary in terms of geometry and particle size. Table 2 shows the specification of the filler systems in terms of the density, the heat capacity (based on own measurements) and the thermal conductivity (based on manufacturer specification). Further, Table 3 depicts the particle geometry based on images taken by a scanning electron microscope (SEM) of the type Gemini Ultra-Plus (Carl Zeiss AG, Oberkochen, Germany). In addition, Figure 2 reveals the particle size in terms of numerical and volumetric counting (A) and the specific surface area (B) (based on own measurements) as well as the Mohs hardness (B) (based on manufacturer specification). 

Boron nitride (BN) of the type CFA 50M SFG (3M Deutschland GmbH, Neuss, Germany) reveals a platelet structure with a high specific surface area. It was mainly chosen because of its high thermal conductivity (in-plane direction). Zinc oxide (ZnO) of the type Silatherm Advanced 1438-800 (Quarzwerke GmbH, Frechen, Germany) reveals the highest specific surface area with very narrow particle sizes and a star-like geometry. It is assumed that the fine distribution of the filler might result in an increase in the thermal conductivity within a sample. Further, aluminum oxide (Al_2_O_3_) of the type Silatherm Plus 1432 (Quarzwerke GmbH, Frechen, Germany), which has the same thermal conductivity as ZnO, was chosen in consideration of its sphere geometry and low specific surface area. In addition, aluminum silicate (Al_2_O_3_ + SiO_2_) of the type Silatherm 1360-010 (Quarzwerke GmbH, Frechen, Germany), which reveals the lowest thermal conductivity, was investigated. Similar to BN, it has a platelet structure with a significantly lower specific surface area. The fillers reveal a stiffness within the range of 65 GPa in terms of Al_2_O_3_ and 74 GPa in terms of BN according to the manufacturer´s specification. Thus, mainly the geometry impact is evaluated within this paper. The fillers were chosen in terms of high thermal conductivity in the first place and, further, to cover a broad range of filler sizes and densities. 

### 2.2. Fabrication of the Test Specimens

**Figure 3:**
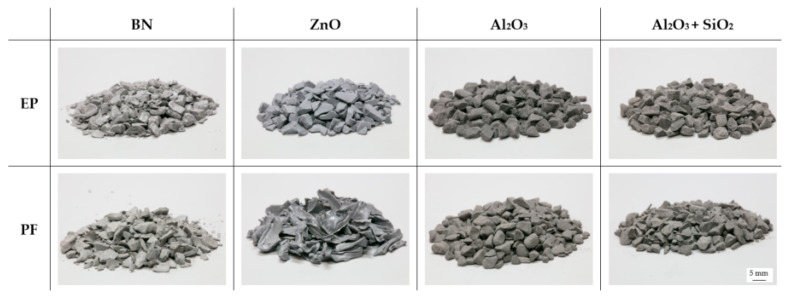
Image of granulates for each material system [representative of the filler grade of 40 vol.-%].

The production of the test specimens was divided into material production by a compounding process and the injection molding of the samples. The matrix and the filler system were mixed manually at room temperature to ensure a filler grade of 40 and 60 vol.-% for each material system. A high-precision weighing device was used to realize the exact proportion of the two components. Further, a homogeneous and sufficient mixing was confirmed by an optical control. By using a twin-screw extruder (type: Kraus Maffei Berstorff ZSE 25Ax45D; KrausMaffei Group, Munich, Germany), the compounds were produced with a screw rotation speed of 80 min^−1^. The temperature was set between 50 °C at the feeding zone and 90 °C at the nozzle. Cooling was realized by a vibratory feeder, followed by pelletizing. Figure 3 reveals the granulates of each material system, representative of the filler grade of 40 vol.-%. 

The samples were fabricated using an injection molding machine (type: KM 80-380 CX DUR/03; Kraus Maffei Group, Munich, Germany) with a screw diameter of 30 mm. In terms of the characterization of the thermal diffusivity, plates were produced with the dimensions of 60 × 60 × 2 [cm^3^]. Further, the mechanical properties were evaluated by using tensile bars. The process parameters were kept as constant as possible. However, some parameters had to be adjusted relative to the geometry of the sample and the material system. Table 4 gives an overview of the process parameters with respect to the different material systems and for both sample geometries. The differences from the reference parameters within each filler system are highlighted in grey. 

### 2.3. Characterization

#### 2.3.1. Differential Scanning Calorimetry (DSC) According to DIN EN ISO 11357

To define the process conditions in terms of the compounding and injection molding process, the temperature-dependent reaction kinetic of the material was characterized using the differential scanning calorimetry (type: DSC 2500; TA instruments, New Castle, DE, USA) under a nitrogen atmosphere according to DIN EN ISO 11357 [13]. Material samples of about 5 mg were heated with a rate of 10 K∙min^−1^ for EP as a matrix material and 5 K∙min^−1^ for PF between the temperatures of 0 and 300 °C. The different heating rates were required in terms of the varying reaction mechanisms in both matrix materials. However, the difference between the heating rates was reduced as much as possible to ensure a comparison of the data. The classification of the DSC measurement was based on the first heating cycle and, thus, on the reaction turnover α, which represents the ratio of the specific enthalpy at the temperature level T_j_ (ΔH_j_) and the total specific enthalpy (ΔH_total;1_). 

#### 2.3.2. Density ρ

To define the density of the fabricated samples, by using the plates, test parts with the dimensions of 10 × 10 × 2 [cm^3^] were prepared out of the middle of a plate by using a water-cooled saw to reduce the temperature impact. The dimensions of the test parts were defined by a digital caliper with a resolution of 0.01 mm. The weight was estimated by a high-precision weighing device. The density was calculated by the ratio of the weight and the volume. Further, the density was compared to the theoretical, calculated value from the density of the pure material components (with respect to Table 1 and Table 2). In addition, the density is needed to calculate the thermal conductivity (in Section 2.3.5) 

#### 2.3.3. Specific Heat Capacity c

The specific heat capacity is another value that is required to calculate the thermal conductivity. The specific heat capacity was defined at 25 °C by using a C80 calorimeter (type: 3D-Calvet calorimeter; TA Instruments, New Castle, DE, USA). Both the pure materials and the compound were analyzed in order to compare the measured values in terms of the compound with the theoretical, calculated ones. Here, the mixing rule defines the specific heat capacity of the compound relative to the mass percentage of the components. The specific heat capacity is temperature-dependent; considering that the application of the material with respect to the focus of the paper is mainly at room temperature, further calculation of the value at different temperatures was not realized. 

#### 2.3.4. Thermal Diffusivity a According to DIN EN ISO 22007

The directionally dependent thermal diffusivity was defined in the x- and z-directions and, thus, parallel and perpendicular to the flow direction using a nanoflash device (type: LFA447; Netzsch GmbH, Selb, Germany) according to DIN EN ISO 22007 [14]. The measurement was held at 23 °C, and a 2 mm broad shutter was used, which was fully splashed. The measurement time was defined as 20,000 s for each spot. In terms of the z-direction (perpendicular to the flow direction), 25 spots were calculated with an even distribution across the plate, and 5 spots were set in height and width directions. In terms of the x-direction (parallel to the flow direction), 5 strips of 3 mm width were prepared by using a water-cooled saw. Again, 5 spots were measured to ensure the same number and position of measurement spots relative to the z-direction. Within this paper, the average of all measurement points was calculated in each direction because the distribution across the plate is not focus of this paper. 

#### 2.3.5. Thermal Conductivity λ According to DIN EN ISO 22007

To compare the results of the thermal diffusivity (in Section 2.3.4) with the reference data given in the literature, the thermal conductivity was calculated by the product of the density, the specific heat capacity and the thermal diffusivity. Further, the ideal values of the density were used within the calculation to understand the impact of the optimized process in detail. 

#### 2.3.6. Mechanical Properties According to DIN EN ISO 527 

The mechanical properties were defined using the tensile bars and a universal tensile testing machine (type: 1464; ZwickRoell GmbH & Co. KG, Ulm, Germany) according to DIN EN ISO 527 [15]. The properties were determined at 23 °C with a traverse speed of 1 mm∙min^−1^ in terms of the modulus and the testing. Besides the stiffness represented by the E-Modulus E_t_, the tensile strength σ_m_ and the elongation at break ε_m_ were defined. 

#### 2.3.7. Filler Distribution and Linkage to Matrix Material 

To analyze the filler distribution in the plates, small strips with a width of 3 mm were prepared along the flow direction of the plate in the middle of the gating system using a water-cooled saw to reduce the temperature impact. The parts were embedded in cold-curing epoxy resin (type: Epofix; Struers GmbH, Ottensoos, Germany) and polished. Images were taken by a stereo microscope (type: Axio Zoom.V16; Carl Zeiss AG, Oberkochen, Germany) with a magnification of 50 for the two filler types Al_2_O_3_ and Al_2_O_3_ + SiO_2_. For the filler types BN and ZnO, images were taken by a scanning electron microscope (type: Gemini Ultra-Plus; Carl Zeiss AG, Oberkochen, Germany) because of the low filler size. Therefore, a 10 nm layer of spray gold was applied. The magnification was 100 for BN and 2500 for ZnO. 

To improve the understanding of the filler distribution and mainly the orientation of the fillers within the plates, the orientation of the fillers was defined quantitatively. Therefore, the angle between the longest axis in a filler and the horizontal was determined, and the main orientation angles were specified between 0° and 90°. This was only possible in terms of the platelet structures of the fillers and, thus, in terms of BN and Al_2_O_3_ + SiO_2_. 

In addition, the linkage between the filler and the matrix material in terms of the tensile bars was defined by using the scanning electron microscope. The magnification was chosen as 500, but it was 5000 for ZnO. 

## 3. Results and Discussion 

### 3.1. Differential Scanning Calorimetry (DSC) According to DIN EN ISO 11357

**Figure 4:**
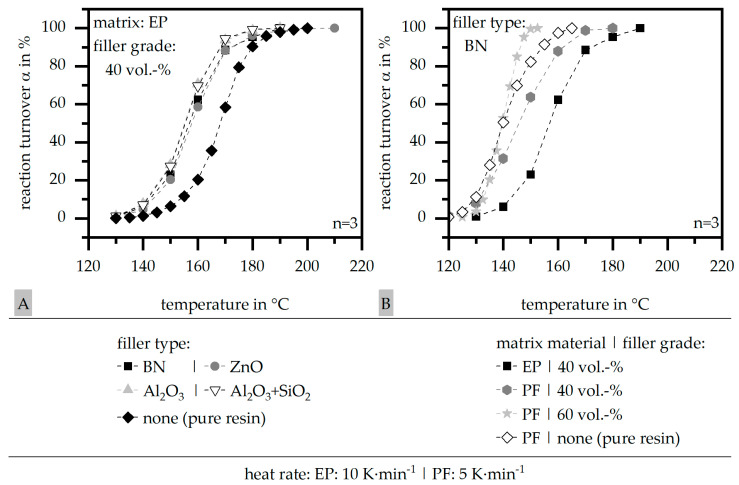
Reaction turnover α relative to the temperature based on DSC measurements relative to the influence of the filler system [representative of EP as matrix material and 40 vol.-% filler grade] (**A**) and the influence of the matrix material and the filler grade [representative of filler system BN] (**B**) as well as the comparison to the pure resin.

The reaction turnover α relative to the temperature is depicted in Figure 4A in terms of the influence of the filler system with a constant matrix material (EP) and a filler grade of 40 vol.-%. Figure 4B further shows the influence of the filler grade (40|60 vol.-%) and the matrix material (EP|PF) for the constant filler type of BN. Further, the behavior is compared to the pure matrix material. The other materials behave similarly. The standard deviation is mostly quite low, so it is not depicted in Figure 4. 

In terms of EP, the reaction turnover α is shifted to lower temperatures in terms of a filled system. The difference between the four filler types is negligible. Despite the different heat rates in terms of the two matrices, the reaction turnover α of PF starts at a lower temperature relative to pure and filled EP, so the fillers shift the reaction turnover α towards higher temperatures in terms of filled PF systems. The increase in the filler grade shifts the reaction turnover α back to lower temperatures. It has to be taken into account that, especially in terms of pure PF, the standard deviation is high due to the reaction product of water, which occurs during the curing and interferes with the measurement. 

### 3.2. Density ρ

**Figure 5:**
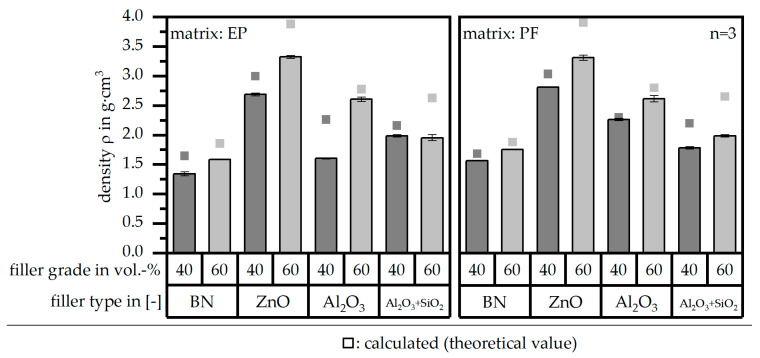
Density ρ of the compound material relative to the filler type and grade as well as the matrix material and in comparison to the theoretical (calculated) value.

Figure 5 shows the density *ρ* of both matrix materials [EP|PF] within the compound relative to the four different filler types and the filler grade of 40 and 60 vol.-%. Further, the measured value is compared to the calculated one. It can be seen that the measured density *ρ* is lower than the theoretical value. The difference between the measured and theoretical value is low for the filler type BN and high for ZnO and Al_2_O_3_ + SiO_2_ for 60 vol.-%. Because the standard deviation is quite low for the measured density *ρ*, the difference between the measured and calculated values is a hint for further improvement in terms of the fabrication of the samples to increase the density *ρ* of the specimens mainly in terms of fewer air inclusions. 

### 3.3. Specific Heat Capacity c

**Figure 6:**
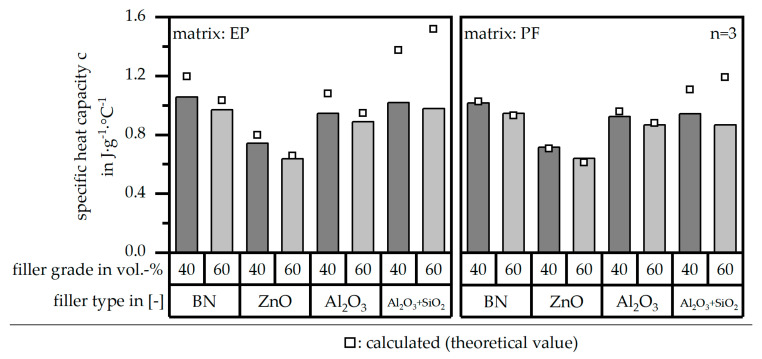
Specific heat capacity c of the compound material relative to the filler type and grade as well as the matrix material and in comparison to the theoretical (calculated) value.

The specific heat capacity c in terms of both matrix materials [EP|PF] and relative to the different filler types and grades is depicted in Figure 6 in comparison to the theoretical values. With the exception of the filler type Al_2_O_3_ + SiO_2_, the measured and calculated values coincide. BN reveals the highest specific heat capacity c values, and ZnO reveals the lowest ones. Both Al_2_O_3_ and Al_2_O_3_ + SiO_2_ reveal similar levels of c. The value of c is decreased in terms of a filler grade independent of the filler system because the filler itself is hardly integrated in the curing process; thus, mainly the matrix amount defines the value of the specific heat capacity c. 

### 3.4. Thermal Conductivity λ According to DIN EN ISO 22007

**Figure 7:**
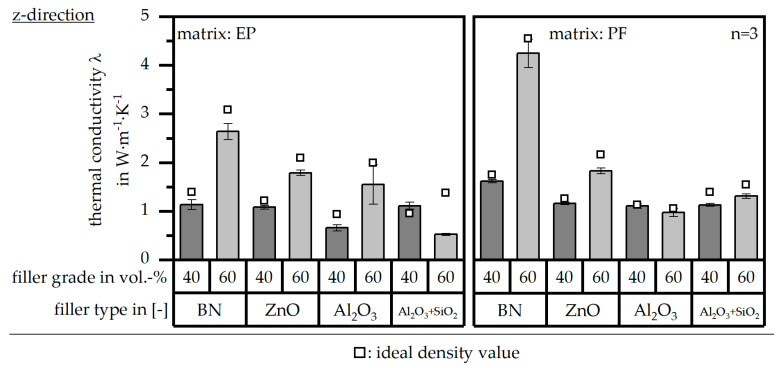
Thermal conductivity λ of the compound material relative to the filler type and grade as well as the matrix material and in comparison to the theoretical value with ideal density in the z-direction (perpendicular to the flow direction).

**Figure 8:**
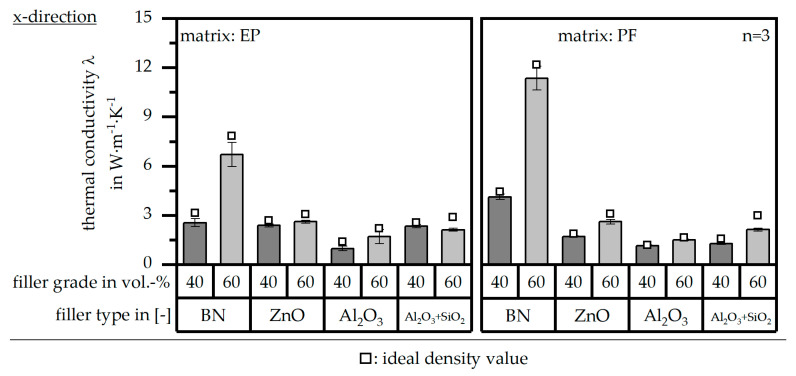
Thermal conductivity λ of the compound material relative to the filler type and grade as well as the matrix material and in comparison to the theoretical value with ideal density in the x-direction (parallel to the flow direction).

The thermal conductivity *λ* in terms of both matrix materials [EP|PF] and relative to the different filler types and grades is depicted in Figure 7 in terms of the z-direction (perpendicular to the flow direction) and Figure 8 in terms of the x-direction (parallel to the flow direction). Both further show the ideal value of the thermal conductivity *λ* in terms of the ideal density values relative to Figure 5. Relative to the literature, the thermal conductivity should reach at least a value of 10 W∙m^−1^∙K^−1^ [10]. These values are only reached by BN as a filler with a matrix material of PF and a filler grade of 60 vol.-% in the x-direction. Overall, the material system based on the filler type BN reaches the highest value in the x- and z-directions. Further, there is a strong anisotropy in terms of the thermal conductivity *λ* for BN, which cannot be seen for the other filler types. It can be seen that the values slightly increase in terms of the ideal value of the density *ρ*. However, the impact of optimized process conditions is rather low. The high values in terms of BN might go along with the platelet structure of the pure filler and the possibility of reaching a homogeneous orientation parallel to the flow direction. This leads to heat paths throughout the sample, and thus, a high thermal conductivity can be reached, as shown in Figure 1. 

### 3.5. Mechanical Properties According to DIN EN ISO 527 

In the following, the results of the mechanical characterization are shown. The results are compared to the values of the pure resin and of industrial standard, as explained in detail in Section 1.3.

**Figure 9:**
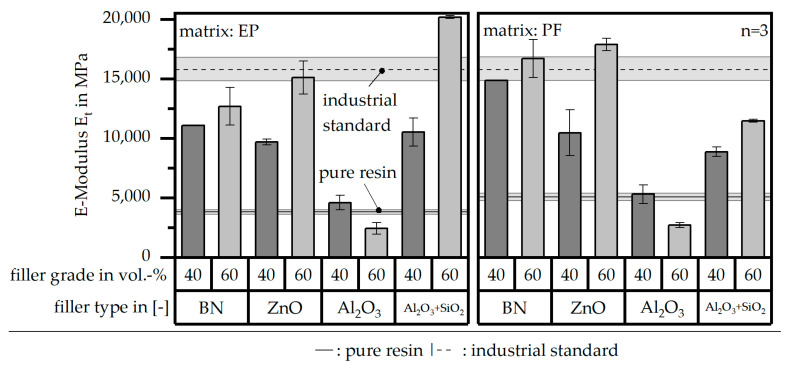
E-Modulus E_t_ of the compound material relative to the filler type and grade as well as the matrix material and in comparison to the values of the pure resin and the industrial standard.

Figure 9 depicts the E-Modulus E_t_ of the different material systems relative to the two references of the pure resin and the industrial standard. All materials except for the filler system Al_2_O_3_ reach higher values compared to the pure resin. However, only the filler type Al_2_O_3_ + SiO_2_ within the EP matrix with a grade of 60 vol.-% and BN as well as ZnO with PF as a matrix again with the grade of 60 vol.-% reach at least the same level as the industrial standard. Except for in the case of the filler Al_2_O_3_, the E-Modulus E_t_ increases with higher filler grades. 

**Figure 10:**
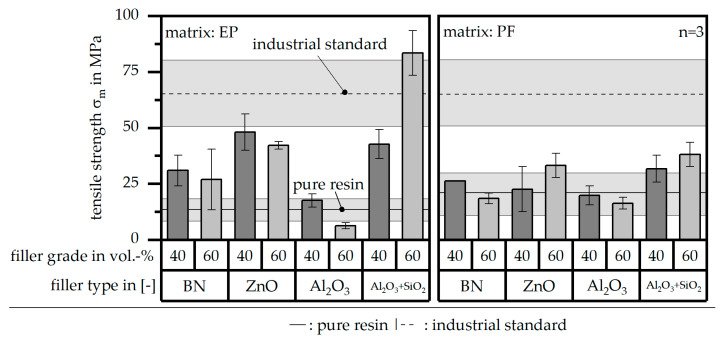
Tensile strength σ_m_ of the compound material relative to the filler type and grade as well as the matrix material and in comparison to the values of the pure resin and the industrial standard.

The tensile strength σ_m_ in terms of the different material systems and relative to the two reference values is depicted in Figure 10. The standard deviation of the pure resin, the compounds and especially the industrial standard is very high. In terms of the matrix material PF, the compounds reveal almost the same values of the tensile strength σ_m_ in the compound relative to the pure resin. In terms of the matrix material EP, only the filler type Al_2_O_3_ + SiO_2_ with a grade of 60 vol.-% reaches the industrial standard. Due to the high standard deviation, a precise perception cannot be realized. 

**Figure 11:**
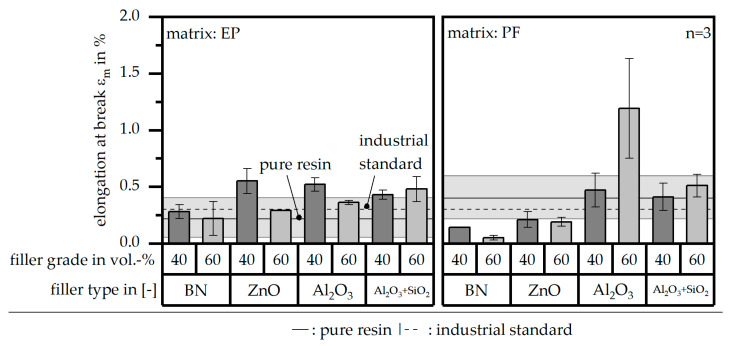
Elongation at break ε_m_ of the compound material relative to the filler type and grade as well as the matrix material and in comparison to the values of the pure resin and the industrial standard.

In addition to the other two properties (E_t_ and σ_m_), the mechanical properties are defined by the elongation at break ε_m_, shown in Figure 11 relative to the material system and the reference values. The industrial standard reveals hardly any standard deviation, whereas the value of the pure resin shows a high deviation. In terms of EP as a matrix material, all compound systems reach at least the industrial standard, except for the filler type BN. In terms of the matrix material PF, the industrial standard is reached by the filler system of Al_2_O_3_ and Al_2_O_3_ + SiO_2_. Further, Al_2_O_3_ with a filler grade of 60 vol.-% within the PF matrix reveals a significantly higher value compared to the other material systems and the reference. However, the standard deviation is quite high as well, which reduces the significant difference compared to the other values slightly. 

In an evaluation of the mechanical results in total, only the material system based on EP as the matrix material and Al_2_O_3_ + SiO_2_ as the filler type with a filler grade of 60 vol.-% gains values that reach or overcome the industrial standard. 

### 3.6. Filler Distribution and Linkage to Matrix Material 

**Figure 12:**
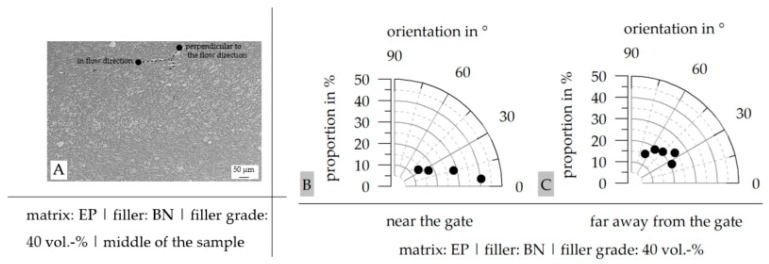
Image of the filler orientation in the middle of the sample (**A**) and orientation of the fillers along the flow direction (0°) near (**B**) and far away (**C**) from the gate for the material EP as a matrix material with 40 vol.-% BN as a filler.

In general, the filler distribution and orientation are increased for the matrix material PF compared to EP and with rising filler grade. The goal in terms of the filler system is the increase in contact points between the fillers to ensure a continuous flow of heat throughout the material. To reach this goal, as shown in the literature (Section 1.3), the fillers should be orientated and reach a high aspect ratio and high sizes. BN reaches most of these criteria in order to increase the path length along the fillers and, with that, the thermal conductivity λ, especially parallel to the flow direction. As shown in Figure 12A, the fillers are orientated parallel to the flow direction and reveal a short distance between individual fillers along the flow path. However, perpendicular to the flow direction, the fillers are orientated as well, but the distance is higher. This is exemplarily shown in Figure 12A. Further, the orientation is depicted in Figure 12B,C for areas near and far away from the gate. Towards the end of the sample (far away from the gate), the orientation decreases.

**Figure 13:**
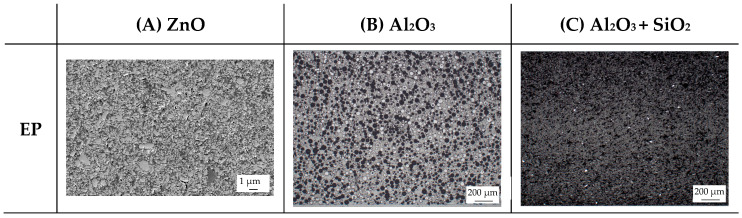
Image of the filler orientation in the middle of the sample, exemplarily shown for the matrix material EP and the filler grade of 40 vol.-% for the filler system ZnO (**A**), Al_2_O_3_ (**B**) and Al_2_O_3_ + SiO_2_ (**C**).

The other three filler systems only partly depict the realization of the three main aspects for increasing the contact area. ZnO loses its geometry, which leads to very small parts of fillers throughout the matrix material. As a result, the gaps between individual filler fragments are small. However, the filler size itself is too small anyhow. This is shown exemplarily in Figure 13A. Further, Al_2_O_3_ (Figure 13B) does not allow an orientation due to the geometry of the filler. Because the aspect ratio is low, the potential of a high contact area is low as well. The filler system Al_2_O_3_ + SiO_2_ has a similar filler geometry compared to BN. However, the orientation is low, as shown in Figure 13C. With respect to the orientation and the images taken, only BN as a filler system reveals the possibility of reaching broad contact areas and, thus, the successful realization of high thermal conductivity.

The linkage between the filler and the matrix material was evaluated in terms of the tested tensile bars and with respect to the fracture surface. Table 5 depicts the images relative to the matrix material and the filler system, representative of a filler grade of 60 vol.-%. It can be clearly seen that the platelet fillers (BN|Al_2_O_3_ + SiO_2_) are more likely to be integrated into the matrix material compared to the other two filler systems. Especially for Al_2_O_3_, the linkage between the filler and the matrix material is rather low. For the filler Al_2_O_3_ + SiO_2_, the matrix material plays an important role because the linkage seems to be higher for EP compared to PF due to an increasing number of negative forms for PF, where the filler was extracted during the cracking. This gives a hint as to why the mechanical properties of EP and the filler system Al_2_O_3_ + SiO_2_ are better than those of the other material systems. Although BN reveals a good linkage, the plates of the filler seem to be stacked, which reduced the mechanical properties due to the sliding of the stacked fillers during the cracking, which is comparable with slate slabs. 

## 4. Discussion

The experiments were based on the objective of increasing both the thermal conductivity λ and the mechanical properties in a thermoset-based highly filled material system. In terms of the mechanical properties, an industrial standard that should at least be reached was defined. In terms of the thermal conductivity λ, only BN shows a significant increase in the values due to the filler, especially for PF as a matrix material and a filler grade of 60 vol.-%. The images taken using a stereo microscope and a scanning electron microscope clearly show that the fillers are orientated in the flow direction for BN, which leads to a high contact area and, with that, an increase in the thermal conductivity λ along the flow path. In comparison to Al_2_O_3_ + SiO_2_, which reveals the same platelet geometry of the filler, the orientation is significantly increased with BN. Because the linkage to the matrix and the filler size are similar, the impact of the fillers on each other, as seen as a sliding effect in terms of the mechanical properties, might be the reason for the better orientation found for BN and, with that, the massive increase in terms of the thermal conductivity λ. 

In terms of mechanical properties, only the material based on Al_2_O_3_ + SiO_2_ with a PF matrix reaches the industrial standard. The filler system BN is near the standard, except for the case of the tensile strength σ_m_, where BN reaches much lower values. This leads to a conflict of objectives because only one of the two criteria can be improved. It is assumed that a slight motivation in terms of the BN-based material system is a promising approach because the sliding effect is important in terms of the thermal conductivity λ and depends on the general geometry of the filler. Therefore, further attempts will be made by the authors to increase the mechanical properties of the BN-based material systems with additives. In addition, the industrial standard as a reference for evaluating the mechanical properties should be examined because these values highly depend on the application. 

## 5. Conclusions

In this paper, four different filler systems and two matrix materials as well as filler grades were combined in order to improve both the mechanical properties and the thermal conductivity λ. It was shown that BN-based material systems reach the highest λ, while Al_2_O_3_ + SiO_2_ is the only filler that allows the mechanical properties given by the industrial standard to be achieved. BN as a filler system is rated as the most promising attempt to reach both criteria due to the addition of additives that improve mechanical properties. 

## Figures and Tables

**Table 1:** Specification of the matrix material including density δ, heat capacity c (own measurements) and thermal conductivity λ as well as molding shrinkage (longitudinal or longwise) (manufacturer specification).

Matrix Material	Density δ in g∙cm^−3^	Heat Capacity c in J∙g^−1^∙°C^−1^	Thermal Conductivity λ in W∙m^−1^∙K^−1^	Molding Shrinkage (Longwise) in %
epoxy resin (EP)	1.2250	1.616	0.4–0.6	0.4–0.6
phenolic resin (PF)	1.2915	1.294	0.4–0.6	0.3–0.5

**Table 2:** Specification of the filler materials including density, heat capacity c (own measurements) and thermal conductivity λ (manufacturer’s specification).

Filler System	Density δ in g∙cm^−3^	Heat Capacity c in J∙g^−1^∙°C^−1^	Thermal Conductivity λ in W∙m^−1^∙K^−1^
boron nitride (BN)	2.27	0.794	15⊥; 400 ∥
zinc oxide (ZnO)	5.65	0.505	30
aluminum oxide (Al_2_O_3_)	3.81	0.785	30
aluminum silicate (Al_2_O_3_ + SiO_2_)	3.56	0.765	14

**Table 3:** Specification of the filler systems including the particle geometry (based on own measurements).

Filler System	BN	ZnO	Al_2_O_3_	Al_2_O_3_ + SiO_2_
particle geometry	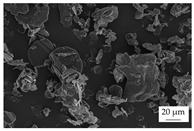	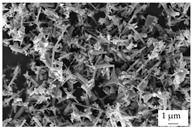	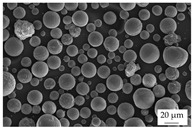	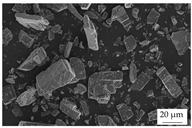
	platelet	star	sphere	platelet

**Table 4:** Process parameters of the fabrication of test specimens (p: plates; t: tensile bars) relative to the material system [grey highlights: difference from reference parameters relative to each filler system].

matrix material	EP		EP		PF	PF	EP	EP	PF	PF	EP	EP	PF	PF		EP	EP	PF	PF	
filler system	BN		BN		BN	BN	ZnO	ZnO	ZnO	ZnO	Al_2_O_3_	Al_2_O_3_	Al_2_O_3_	Al_2_O_3_		Al_2_O_3_ + SiO_3_	Al_2_O_3_ + SiO_3_	Al_2_O_3_ + SiO_3_	Al_2_O_3_ + SiO_3_	
filler grade	40		60		40	60	40	60	40	60	40	60	40	60		40	60	40	60	
sample type	p	t	p	t										p	t				p	t
**process parameter**																				
mass temperature T_m_ in °C [feeding|noozle]	55|95	75|110	55|95	75|110	55|95	55|105	65|85	65|85	65|85	65|95	65|95	65|100	65|85	65|105	65|85	55|110	55|85	55|85	65|85	75|115
mould temperature T_WZ_ in °C	160	180	160	180	160	160	180	180	180	180	180	180	180	180	180	160	160	160	160	160
heating time t_h_ in s	85	85	85	85	75	75	75	75	40	40	85	85	30	30	30	75	75	30	30	30
injection speed V_m_ in mm s^−1^	15	15	15	15	15	15	15	15	15	15	15	15	15	15	15	15	25	25	15	15

**Table 5:** Linkage between the filler and the matrix material relative to the tensile bars [matrix material: EP|PF; four different filler systems; filler grade: 60 vol.-%].

	BN	ZnO	Al_2_O_3_	Al_2_O_3_ + SiO_2_
**EP**	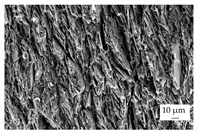	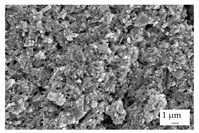	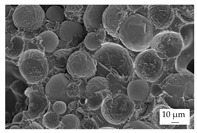	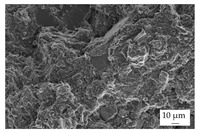
**PF**	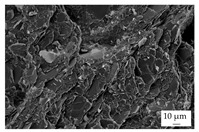	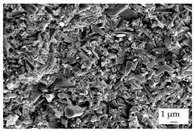	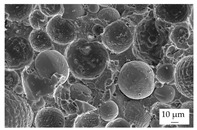	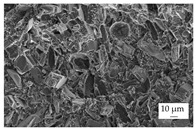

## Data Availability

Restrictions apply to the availability of these data. Data are available with the permission of the author.
